# The Draft Genome Dataset of the Asian Cricket *Teleogryllus occipitalis* for Molecular Research Toward Entomophagy

**DOI:** 10.3389/fgene.2020.00470

**Published:** 2020-05-08

**Authors:** Kosuke Kataoka, Ryuhei Minei, Keigo Ide, Atsushi Ogura, Haruko Takeyama, Makio Takeda, Takeshi Suzuki, Kei Yura, Toru Asahi

**Affiliations:** ^1^School of Advanced Science and Engineering, Waseda University, Tokyo, Japan; ^2^Department of BioScience, Nagahama Institute of Bio-Science and Technology, Shiga, Japan; ^3^Computational Bio Big-Data Open Innovation Laboratory (CBBD-OIL), National Institute of Advanced Industrial Science and Technology, Tokyo, Japan; ^4^Global Consolidated Research Institute for Science Wisdom, Waseda University, Tokyo, Japan; ^5^Institute for Advanced Research of Biosystem Dynamics, Waseda Research Institute for Science and Engineering, Waseda University, Tokyo, Japan; ^6^Research Organization for Nano & Life Innovation, Waseda University, Tokyo, Japan; ^7^Department of Agrobioscience, Graduate School of Agricultural Science, Kobe University, Hyogo, Japan; ^8^Graduate School of Bio-Applications and Systems Engineering, Tokyo University of Agriculture and Technology, Tokyo, Japan; ^9^Graduate School of Humanities and Sciences, Ochanomizu University, Tokyo, Japan

**Keywords:** draft genome, hybrid assembly, edible crickets, mitochondrial genome, *Teleogryllus*

## Introduction

The world population is predicted to steadily rise to nine billion by 2050 and it requires the increase of food production by 70% (Godfray et al., 2010). However, improvement in food production efficiency is not expected, due to the threat by rapid urbanization, water shortages, overfishing, climate change, and land degradation (Nellemann et al., 2009). Additionally, the present food industry dumps diverse wastes that are poorly reused (Brosowski et al., 2016). To overcome these challenges, insects have emerged as one of the sustainable alternative protein sources for humans and livestock. Insects can utilize water and food more efficiently, showing lower feed conversion rate and better growth efficiency when compared with conventional livestock (Nagasaki and Defoliart, 1991; Oonincx et al., 2015). Crickets, for example, consume six and three times less feed than cattle and pigs, respectively, to produce the same amount of protein (Nagasaki and Defoliart, 1991). In addition, crickets emit less greenhouse gases and ammonia than conventional livestock, and can be reared on organic side-streams such as food waste or by-products from agriculture and food industry (Oonincx et al., 2010, 2015; Cičková et al., 2015). In spite of these advantages, insects have not been utilized for food and feed yet, because insect farming is believed to be not cost-effective. Therefore, highly efficient harvesting, mass-production system, and selective breeding are in need to assure a safe, sustainable, and reliable insect production.

Orthoptera including locusts and crickets are commonly and frequently consumed and reared around the world. In Asia, crickets, such as *Teleogryllus occipitalis, T. mitratus, Acheta domesticus*, and *Gryllus bimaculatus*, are harvested from the wild or reared as edible insects (van Huis et al., 2013). As of 2012, ~20,000 farmers reared crickets in Thailand (van Huis et al., 2013). Even in Europe and North America, the market for cricket-based products, such as protein powder, granola bars, crackers, or cookies, is rapidly growing. Additionally, the nutrition of crickets is rich in beneficial and essential amino acids, lipids, and fatty acids, which is enough for feeding fish and livestock (Wang et al., 2004; Yang et al., 2006). Although crickets are being promoted as new nutrient sources for animals and even humans, further development of rearing, breeding, and processing is required for industrial mass production of crickets. However, the development of industrial cricket rearing is hampered partly by the lack of reference genome; there is currently no dataset for draft genome and annotation set for industrial crickets.

### Value of Data

The industry of farming crickets (Orthoptera: Gryllidae) is currently growing in a global market to supply one of the most promising alternative sources of protein for human food and animal feed.

The hybrid Illumina/Nanopore sequencing and assembly produced a draft genome and annotations.

This dataset will contribute to molecular research and more efficient targeted genetic intervention for industrial mass production of crickets.

## Materials and Methods

### Sample Collection and DNA/RNA Extraction

**Figure 1:**
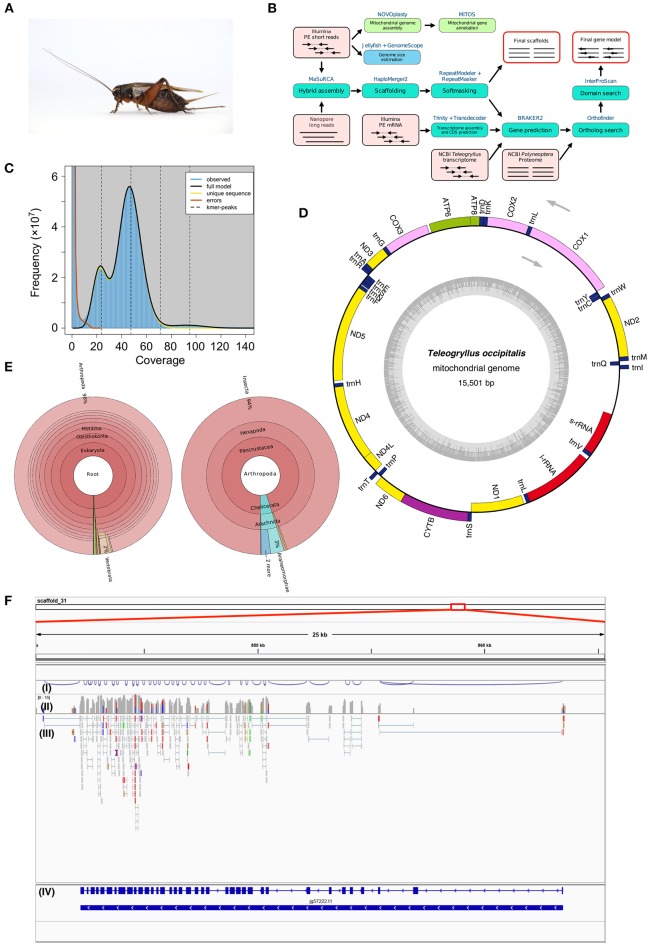
**(A)** Female adult *Teleogryllus occipitalis*. Live specimen was anesthetized by carbon dioxide and pictured. **(B)** *Teleogryllus occipitalis* genome assembly and annotation pipeline. PE, paired-end. The figure shows inputs (light pink boxes), processes (blue or green boxes), and outputs (red outline). **(C)** The *k*-mer distribution (*k* = 21) of *Teleogryllus occipitalis*. The 21-mer distribution was calculated by GenomeScope based on 139.0 Gbp Illumina short reads data (insert size = 200 bp). *K*-mer coverage (*x* axis) were plotted against their frequencies (*y* axis). The left and right peaks of the full model correspond to the heterozygous and homozygous peaks, respectively. **(D)** The complete mitochondrial genome map of *Teleogryllus occipitalis*. 13 protein-coding genes, 2 ribosomal RNA genes (s-rRNA [12S ribosomal RNA] and l-rRNA [16S ribosomal RNA]), 22 transfer RNA (tRNA) genes were annotated and depicted in this map. The inner circle represents GC% along the genome sequence. **(E)** Krona chart representing taxonomic composition of *Teleogryllus occipitalis* gene model. Taxonomy charts, which consist of all taxa (left) and subphylums of Arthropoda (right), are shown. **(F)** Gene structure and gene expression of *Vitellogenin receptor* in *Teleogryllus occipitalis* visualized by IGV genome viewer. (I) Visualization of splicing junctions, (II) Coverage of RNA-seq data for Illumina short reads, (III) Mapping of RNA-seq data for Illumina short reads, and (IV) Gene structure of putative *Vitellogenin receptor*.

*T. occipitalis* was collected in Amami Oshima Island, Kagoshima, Japan, and reared and inbred in the laboratory (M.T.). For genome sequencing using Illumina, head, and femur were dissected from single female individual (Figure 1A), while, for Nanopore long-read and RNA sequencing, muscle tissues were dissected from the femur of which exoskeletons were removed from multiple mix-gender individuals. These samples were lysed in an SDS-containing lysis buffer (50 ml Tris-HCl (pH 8.0), 4 mM NaCl, 20 mM EDTA, 1% SDS) overnight together with 0.2 mg/ml proteinase K at 55°C. For DNA and RNA purification, the homogenates were incubated with RNase and DNase, respectively, for 30 min at 37°C. An equal amount volume of Tris-saturated phenol (pH 8.0) was added, mixed, and centrifuged at 13,200 × *g* for 3min, then the supernatant was collected and transferred to a new tube. This extraction process was repeated using chloroform. 1/5 volume of 5 M NaCl and equal amount volume of absolute ethanol were added to the supernatant, followed by centrifugation at 13,200 × *g* for 5 min. The resulting precipitate was rinsed with 70% ethanol, dried, dissolved with TE buffer (10 mM Tris-HCl (pH 8.0), 1 mM EDTA) and stored at 4 or −80°C for DNA and RNA, respectively, until further processing.

### Library Preparation and Sequencing

Extracted genomic DNA and total RNA of *T. occipitalis* were shipped to Macrogen Japan (Kyoto, Japan) for library preparation and sequencing. These experiments were performed based on the manufacturer's protocol. For DNA-seq, the sequencing libraries were prepared using TruSeq DNA PCR-Free kit (Illumina, San Diego, CA, USA), and the prepared libraries were sequenced by HiSeq X. In RNA-seq, the library was prepared using TruSeq stranded mRNA kit and sequenced by NovaSeq 6000. Hiseq X produced 139 Gbp derived from 2 × 150 paired-end reads, which were ~71.67 X coverage of *T. occipitalis* genome. NovaSeq 6000 produced 4.3 Gbp from 2 × 100 paired-end reads.

For long-read sequencing using MinION (Oxford Nanopore Technology, Oxford, UK), the extracted genomic DNA with high quality was fragmented to ~20 Kbp using Covaris g-TUBE (Covaris, Woburn, MA, USA). After purification using 0.4 × AMPure XP beads (Beckman Coulter, Brea, CA, USA), the library was prepared using the SQK-LSK109 Ligation Sequencing kit (Oxford Nanopore Technologies) based on the manufacturer's protocol. The prepared library was loaded onto R9.4.1 chemistry flowcell (FLO-MIN106) and sequenced using MinKNOW (v1.19.06). After sequencing, Guppy v3.2.1 was used for basecalling, finally resulting in 32.40 Gbp long read data, which were 16.76 X coverage with N50 of 8.068 Kbp. The statistics of sequencing results are summarized in Table S1.

### Genome Assembly

The genome assembly and annotation pipeline are shown in Figure 1B. We estimated the overall characteristics of *T. occipitalis* genome, such as its genome size, heterozygosity, and repeat content, by *k*-mer frequencies calculated from Illumina short reads. Jellyfish v2.2.10 (Marçais and Kingsford, 2011) was used to obtain 21-mer count histogram of the short DNA reads. GenomeScope v1.0.0 (Vurture et al., 2017), *k*-mer analysis software, estimated the genome size of about 1.87 Gbp (Figure 1C), which was almost comparable with that of our scaffolded draft genome (1.93 Gbp). The genome of crickets that have been already reported [*T. oceanicus* (Pascoal et al., 2019) and *Laupala kohalensis* (Blankers et al., 2018)] have the size of about 2.05 and 1.60 Gbp, respectively, which are similar to our *T. occipitalis* draft genome size.

To obtain the draft genome of *T. occipitalis* with high quality, we applied the technique called the hybrid assembly using Illumina short-reads and Nanopore long-reads. Short and long reads were assembled to contigs using MaSuRCA assembler v3.3.0 (Zimin et al., 2017). For gap-closing, assembled contigs were scaffolded into the draft genome using HaploMerger2 pipeline v3.4 (Huang et al., 2017). The scaffolded draft genome has a genome size of 1.934 Gbp, scaffold number of 19,865, N50 of 214.13 Kbp and the longest scaffold of 2.3 Mbp, as calculated by QUAST v5.0.1 (Gurevich et al., 2013) (Table 1A). We evaluated the gene completeness of our draft genome using BUSCO tool v2/3 (Simão et al., 2015; Waterhouse et al., 2018). BUSCO analysis with Arthropoda datasets revealed 98.1% of complete orthologs (sum of the percentages of single-copy and duplicate), strongly suggesting that our draft genome possessed the sufficient gene repertoire of *T. occipitalis* (Table 1A). The quality of our draft genome of *T. occipitalis* was comparable, or even much better than those of other Orthoptera genomes that have been previously reported (Wang et al., 2014; Blankers et al., 2018; Pascoal et al., 2019).

**Table 1:** Draft Genome and Annotations Statistics **(A)** Scaffold statistics of Orthoptera genomes including our draft genome in this study. **(B)** Statistics of our *T. occipitalis* gene model.

	**Tocc**	**Toce**	**Lkoh**	**Lmig**
**(A) Scaffold statistics**				
Total scaffolds (Total contigs)	19,865 (39,249)	197,895	148,874	254,516
Total scaffold sequence (Gbp)	1.9	2.0	1.6	6.5
Scaffold N50 (Kbp) [Contig N50 (Kbp)]	214 (122)	63	583	730
Longest scaffold (Mbp)	2.3	2.6	4.5	5.2
BUSCO Complete, Single+Duplicate (%)	98.1	93.9	99.3	94.0
**(B) Gene set statistics**				
Number of protein-coding genes	20,768			
Med. CDS length (bp)	996			
Number of single-exon genes	6,029			
Med. exon number per gene	4			
Med. exon length (bp)	160			
Med. intron length (bp)	862.5			
Max. exon number	161			
BUSCO Complete, Single + Duplicate (%)	96.4			

*Tocc, Teleogryllus occipitalis; Toce, Teleogryllus oceanicus; Lkoh, Laupala kohalensis; Lmig, Locusta migratoria*.

Generally, insect genomes are contaminated with intracellular bacteria genomes from Wolbachia for instance. However, a previous study suggested that an organ such as brain and muscle from adult *T. occipitalis* was not suitable for the proliferation of Wolbachia (Kamoda et al., 2000). Therefore, we carefully selected tissues for DNA sequencing to avoid potential Wolbachia contamination. As a consequence, analysis with BBsketch (Bushnell, 2014) against NCBI nt database has revealed that our data contained only a single scaffold with a length of 9,712 bp with a putative Wolbachia sequence. But even in this case, its query coverage was only 8.4% (815 bp) of the scaffold. Evidently, the draft genome sequence obtained in this study is virtually free from bacterial genome contamination.

### Mitochondrial Genome Assembly and Annotation

For mitochondrial genome assembly, after trimming Illumina short-reads with Trimmomatic v0.38 (Bolger et al., 2014), the trimmed reads were assembled by NOVOplasty v3.7.1 (Dierckxsens et al., 2017) with partial CDS of cytochrome oxidase subunit I gene from *T. occipitalis* (GenBank: MF046165.1) as a seed. A resulting single circularized mitochondrial genome was annotated with MITOS2 (Bernt et al., 2013), visualized by OGDRAW v1.3.1 (Greiner et al., 2019), and shown in Figure 1D.

The complete mitochondrial genome of *T. occipitalis* consisted of 15,501 bp, and includes 37 genes: two rRNA genes (l-rRNA, s-rRNA), 22 tRNA genes (Ala, Arg, Asn, Asp, Cys, Gln, Glu, Gly, His, Ile, Leu-1, Leu-2, Met, Lys, Phe, Pro, Ser-1, Ser-2, Thr, Trp, Tyr, Val), and 13 protein-coding genes (*atp6, atp8, cox1, cox2, cox3, cytb, nad1, nad2, nad3, nad4, nad4L, nad5, nad6*). Directions and order of these genes were comparable with those of the previously described complete mitochondrial genome of other *Teleogryllus* species (*T. oceanicus*: NC_028619.1, *T. emma*: NC_011823.1).

### Repeat Analysis

We obtained repetitive element library from the scaffolded genome by *de novo* approach using RepeatModeler v1.0.11 (Smit and Hubley, 2015). This repetitive element library was then utilized by RepeatMasker to annotate repetitive elements. Summary of the annotation is shown in Table S2. The estimated repeat regions accounted for 44.75% of the genome, comprising 31% of unclassified repeat, followed by 7.26% of long interspersed nuclear elements (LINE) (Table S2). Analysis on the unclassified 1,422 repeats using BLASTn against NCBI nt database has revealed that 138 repeats were phylogenetically annotated. Further, 71% of the annotated repeats were taxonomically annotated as *Gryllinae*, indicating that some of the unclassified repeats might be unique sequences in this lineage.

### Annotations

After soft-masking the repeat regions, we predicted genes based on RNA-seq mapping and *de novo* assembled transcriptome data. First, we sequenced and assembled 100 bp paired-end RNA-seq reads using Trinity v2.8.4 (Grabherr et al., 2011) pipeline without any template, following AfterQC v0.9.6 (Chen et al., 2017). This resulted in a transcriptome assembly comprising 57,217 transcripts. 97.01% of the reads were aligned to genome sequence as a result of mapping the reads to scaffolded *T. occipitalis* genome with HISAT2 v2.1.0 (Kim et al., 2015), demonstrating high congruity between RNA-seq and DNA-seq reads. Following the prediction of coding regions with TransDecoder v5.5.0 (Haas et al., 2013) in the assembled transcriptome, the complete, and not partial, coding regions were extracted by in-house script. BRAKER v2.1.4 (Hoff et al., 2016) pipeline was used to predict the genes of *T. occipitalis* with extrinsic evidence of our *T. occipitalis* transcriptomic data together with those of other *Teleogryllus* species, *T. emma*, and *T. oceanicus*, acquired from the public database (SRX3181975-SRX3181978 and ERX2641045-ERX2641056).

We next conducted ortholog analysis using Orthofinder v2.3.3 (Emms and Kelly, 2019) to obtain conserved genes in closely related species and found 13,666 genes which were shared with those of *Cryptotermes secundus, Blattella germanica*, or *Zootermopsis nevadensis* (Proteome ID: UP000235965, UP000245037, UP000027135, respectively). The remaining *T. occipitalis*-specific genes were screened by InterProScan v5.36-75.0 (Jones et al., 2014). At the end, these steps resulted in 20,768 candidate protein-coding gene model. Functional annotation of this gene model was conducted using BLASTp v2.7.1+ (E-value = 1.0 × 10^−5^) (Camacho et al., 2009). As a consequence, 77.5% of genes in our model were annotated against reviewed Swiss-Prot, 69.0% against protein datasets from *Homo sapiens* in UniPort database, and 65.4% against protein datasets from *Drosophila melanogaster* in Uniprot database (Bairoch, 2000). Furthermore, InterProScan v5.36-75.0 (Jones et al., 2014) annotated 95.8% of the total genes. The BUSCO transcriptome analysis with Arthropoda datasets found 96.4% of completeness in the annotation dataset (Table 1B). These results underscore high levels of completeness of the assembly and the gene model of *T. occipitalis* reported here.

We further investigated the closest protein homolog of each entry in the gene model of *T. occipitalis* using NCBI nr dataset with diamond v0.9.24 (*E*-value = 1.0 × 10^−5^), and the result is visualized by Krona (Ondov et al., 2011) (Figure 1E). Approximately 96% of the closest protein homolog of each gene of the gene model belong to Arthropoda. Of the proteins that were detected in Arthropoda, about 94% were derived from Insecta, indicating that the gene model is quite consistent with the taxonomical location of *T. occipitalis*. Interactive Krona viewer is deposited to figshare (Kataoka et al., 2019).

### Gene Structure Analysis

Gene parameters of the gene model in *T. occipitalis* are shown in Table 1B. The medians of CDS, exon and intron lengths, and exon number per gene are comparable to those of closely related species such as *Polyneoptera* (Li et al., 2018).

Validity of the gene structure is exemplified in *Vitellogenin Receptor* (*VgR*), one of the most important genes for fecundity in insects (Tufail and Takeda, 2009; Xu et al., 2010) (Figure 1F). *T. occipitalis* genome contained a single gene for *VgR*, spanning > 21 kb with 37 exons. Note that the intron-exon structure of *VgR* was well supported by RNA-seq data as shown in (III) of Figure 1F [visualized by IGV genome viewer v2.7.2 (Thorvaldsdóttir et al., 2013)]. The predicted VgR protein consists of 1,877 amino acid residues, all of which are consistent with the homologous genes/proteins found in *Blattella germanica* (Ciudad et al., 2006) and *Bombyx mori* (Lin et al., 2013) genome sequences.

## Code Availability

Software used for read preprocessing, genome and transcriptome assembly and annotation is described in the Methods section together with the versions used. Codes used for these processes were deposited on GitHub (https://github.com/MushiHackers/Teleogryllus_occipitalis).

## Data Availability Statement

Raw sequencing libraries, genome assembly, and transcriptome assembly were deposited to NCBI SRA as part of the BioProject PRJDB9056. The raw sequencing reads data were deposited to DNA Data Bank of Japan (DDBJ) Sequence Read Archive (DRA). Illumina DNA-seq, Nanopore DNA-seq, and RNA-seq were available in DRR201339, DRR201340, and DRR201341, respectively. The assembled genome sequence and complete mitochondrial genome sequence were available in DDBJ (BLKR01000001-BLKR01019865 and LC521855, respectively). Datasets for structural and functional annotation are available at figshare (https://doi.org/10.6084/m9.figshare.11474016) (Kataoka et al., 2019).

## Author Contributions

MT established cultures of *T. occipitalis*. KK, RM, and KI conducted sequencing, genome assembly and gene annotation. KK, RM, KI, TS, and KY conducted data analysis and wrote the manuscript. AO, HT, TS, KY, and TA conceived and supervised this study.

## Conflict of Interest

The authors declare that the research was conducted in the absence of any commercial or financial relationships that could be construed as a potential conflict of interest.
