# Supplementary material for: The Draft Genome Dataset of the Asian Cricket Teleogryllus occipitalis for Molecular Research Toward Entomophagy
Source: Front Genet. 2020 May 8;11:470. doi: 10.3389/fgene.2020.00470 (PMC7225344; doi:10.3389/fgene.2020.00470)
Supplement: Supplementary file 1 [file Data_Sheet_1.docx]

Supplementary Material

**Supplementary Table S1.** Sequencing Statistics

| **Illumina paired-end genomic DNA** |  |
| --- | --- |
| Number of reads | 92,020,849 |
| Total library size (Gbp) | 139 |
| Insert size (bp) | 200 |
| Read length (bp) | 2 × 150 |
| Estimated Coverage | 71.67 × |
| **Nanopore genomic DNA** |  |
| Number of reads | 7,024,689 |
| Total library size (Gbp) | 32.4 |
| Read length N50 (Kbp) | 8.068 |
| Estimated Coverage | 16.76 × |
| **Illumina paired-end mRNA** |  |
| Number of reads | 42,927,884 |
| Total library size (Gbp) | 4.336 |
| Read length (bp) | 2 × 100 |

**Supplementary Table S2.** Repeat Analysis

| Repeat elements | Copies | Bases | Percent (%) |
| --- | --- | --- | --- |
| Interspersed repeats | | | |
| SINE | 0 | 0 | 0 |
| LINE | 279,128 | 140,416,137 | 7.26 |
| LTR elements | 12,134 | 15,643,459 | 0.81 |
| DNA elements | 100,209 | 36,252,344 | 1.87 |
| Unclassified | 2,180,281 | 600,024,978 | 31.03 |
| **Total interspersed repeats** | **2,571,752** | **792,336,918** | **40.97** |
| Tandem repeats | | | |
| Satellites | 446 | 388,874 | 0.02 |
| Simple repeats | 815,514 | 63,918,221 | 3.31 |
| Low complexity | 97,817 | 8,685,388 | 0.45 |
| Small RNA | 0 | 0 | 0 |
| **Total tandem repeats** | **913,777** | **72,992,483** | **4** |
| **Total** | **3,485,529** | **865,329,401** | **44.75** |
